# Karyotypic and mtDNA based characterization of Malaysian water buffalo

**DOI:** 10.1186/s12863-019-0741-0

**Published:** 2019-03-25

**Authors:** Nor ‘ Ammar Liyana Shaari, Marilyn Jaoi-Edward, Shu San Loo, Mohd Shahrom Salisi, Rosnina Yusoff, Nurul Izza Ab Ghani, Mohd Zamri Saad, Hafandi Ahmad

**Affiliations:** 1 Department of Veterinary Preclinical Sciences, Faculty of Veterinary Medicine, Universiti Putra Malaysia, 43400 Serdang, Malaysia; 2 Agro-Biotechnology Institute (ABI), National Institutes of Biotechnology Malaysia (NIBM), c/o MARDI Headquarters, 43400 Serdang, Malaysia; 3 Department of Veterinary Clinical Studies, Faculty of Veterinary Medicine, Universiti Putra Malaysia, 43400 Serdang, Malaysia; 4 Department of Biology, Faculty of Science, Universiti Putra Malaysia, 43400 Serdang, Malaysia; 5 Research Centre for Ruminant Diseases, Faculty of Veterinary Medicine, Universiti Putra Malaysia, 43400 Serdang, Malaysia

**Keywords:** Karyotyping, Mitochondrial DNA, Phylogenetic, Water buffaloes

## Abstract

**Background:**

In Malaysia, the domestic water buffaloes (*Bubalus bubalis*) are classified into the swamp and the murrah buffaloes. Identification of these buffaloes is usually made via their phenotypic appearances. This study characterizes the subspecies of water buffaloes using karyotype, molecular and phylogenetic analyses. Blood of 105 buffaloes, phenotypically identified as swamp, murrah and crossbred buffaloes were cultured, terminated and harvested using conventional karyotype protocol to determine the number of chromosomes. Then, the D-loop of mitochondrial DNA of 10 swamp, 6 crossbred and 4 murrah buffaloes which were identified earlier by karyotyping were used to construct a phylogenetic tree was constructed.

**Results:**

Karyotypic analysis confirmed that all 93 animals phenotypically identified as swamp buffaloes with 48 chromosomes, all 7 as crossbreds with 49 chromosomes, and all 5 as murrah buffaloes with 50 chromosomes. The D-loop of mitochondrial DNA analysis showed that 10 haplotypes were observed with haplotype diversity of 0.8000 ± 0.089. Sequence characterization revealed 72 variables sites in which 67 were parsimony informative sites with sequence diversity of 0.01906. The swamp and murrah buffaloes clearly formed 2 different clades in the phylogenetic tree, indicating clear maternal divergence from each other. The crossbreds were grouped within the swamp buffalo clade, indicating the dominant maternal swamp buffalo gene in the crossbreds.

**Conclusion:**

Thus, the karyotyping could be used to differentiate the water buffaloes while genotypic analysis could be used to characterize the water buffaloes and their crossbreds.

## Background

Water buffaloes, *Bubalus bubalis* are domesticated animals that play essential roles in agriculture, economy and food production. Water buffaloes are distributed widely around the world including the Indian subcontinent, Southeast Asia, China and across continents in Italy and Australia [1, 2]. In Malaysia, water buffaloes have great potential for meat and milk productions, especially in Sabah and a few states in Peninsular Malaysia [3–5].

The domestic water buffaloes are classified into two groups, based on their morphological and ecological characteristics. They are the swamp, *Bubalus bubalis carabensis* and the murrah, *Bubalus bubalis bubalis* buffaloes. In agriculture, swamp buffaloes are reared mainly for draught power in the paddy fields, oil palm and rubber plantations [6], while the murrah buffaloes produce good quality milk [7]. Morphologically, swamp buffaloes are small in size and tend to wallow in murky water such as swamp [8]. However, the murrah buffaloes are large in size with prominent horn and black jet in colour. In addition, murrah or “river” buffaloes prefer wallowing in clean water such as river, thus giving them the name [1]. Mostly, identification of the two subspecies of buffaloes is conducted based on conventional technique such as the morphological or physiological characteristics. Nevertheless, genetic predisposition, dominance and origin could not be determined using the conventional technique, particularly on crossbreds and effects of crossbreeding. Thus, the use of more accurate techniques are important to identify the phylogeny of the animals, or to compare the crossbred with their purebred parents [9, 10]. The techniques include karyotyping and molecular identification using molecular markers.

Karyotyping has been used to identify buffalo species while identification by molecular approach strengthen and supports the findings from the karyotyping. The mitochondrial DNA (mtDNA) is inherited maternally and has been influential for identifying ancestors, localizing domestication centres, tracking colonization and trading routes [11]. Most studies using mtDNA in buffaloes target the variable parts of the control region and the *cytochrome b* gene that look at population and phylogenetic relationships [12]. The D-loop mitochondrial DNA segment has been greatly emphasized because it acts as a crucial tool for assessing genetic relationships of individuals within species or different species. Thus, construction of phylogenetic tree should reveal the closeness of the subspecies of water buffalo. Therefore, the aims of this study were to identify the two subspecies of water buffaloes and their crossbred through karyotype and to use molecular approaches to characterize these buffaloes.

## Methods

### Animals

Water buffaloes (*n* = 105) were randomly selected from a government buffalo breeding farm in Sabah, Borneo and a private buffalo farm in Semenyih, Peninsular Malaysia. The swamp (*n* = 93) and crossbred (*n* = 7) buffaloes were selected from Sabah while the murrah (*n* = 5) buffaloes were selected from Semenyih, Selangor. The selection of each species was based on the phenotypic criteria. Both farms practiced natural breeding while the crossbreeding was done only in the government farm, involving breeding between the murrah males with swamp females.

The selected buffaloes were placed in paddocks for 3 days and were allowed to graze the *Brachiaria* grass (moisture: 6.56%, ash: 5.37%, crude fiber: 26.13%, crude fat: 5.11%, crude protein: 17.12%). Supplemented feed (moisture: 9.64%, ash: 5.44, crude fiber: 7.49, crude fat: 5.46%, crude protein: 18.15%) was provided at the rate of 1.5 kg/animal/day and drinking water was available ad libitum. The buffaloes were monitored daily and all animal handling and techniques were approved by the Institutional for Animal Care and Use Committee (IACUC), Universiti Putra Malaysia (UPM/IACUC/AUP-U017/2018).

### Karyotyping analysis

Approximately 5–8 ml blood was drawn by venepuncture of the tail vein into a vacutainer tube containing heparin. The blood was immediately centrifuged at 1800 rpm for 10 min (Hettich, Zentrifugen D-7200 Tuttlingen) to produce a layer of buffy coat. Then, the buffy coat was pipetted slowly into the culture medium containing 8.0 ml of RPMI 1640, 2.0 ml of bovine calf serum, 0.1 ml of antibiotic, and 0.1 ml of mitogen (Pokeweed, PHA). The mixture was mixed gently and incubated at 37 °C for 72 h, while gently shaken twice daily to prevent leucocytes adhering to the walls of the culture flasks [13].

One hour before harvesting, 0.1 ml of colcemid was added into the culture bottle to stop the multiplying cells at metaphase. The mixture was gently shaken and incubated for another 1 h. At the end of the incubation period, the culture was gently shaken before the suspension was transferred into a 15 ml conical centrifuge tube. The suspension was spun for 5 min at 1800 rpm before the content was taken out leaving only 2.0 ml of the medium. Then, 6.0 ml of pre-warmed (37 °C) 0.075 M potassium chloride was introduced into each tube. The suspension was incubated at 37 °C C for 20 min and then centrifuged for 8 min at 1800 rpm. The supernatant was decanted with 6.0 ml fresh acetic methanol (1, glacial acetic acid: 3 methanol carnoy’s fixative) and was added into individual tube. The suspension was mixed well using pipette. The fixation step was repeated 3 times with the final suspension in 3 ml of carnoy’s fixative. The cell button was gently mixed with pasteur pipette. Two drops of 50% acetic acid were initially placed onto pre-clean slides, followed by another two drops before a drop of the cell suspension was added and spread. The slide was air-dried and another drop was put slightly away from the first. Next, Giemsa stain was used to cover the entire slide, washed with distilled water and air dried before viewing under microscope. The counting of chromosomes was performed on metaphase cells under the light microscope (Motic, China). Ten clearly observable spread of each sample were picked out and photographed (Motic Images Plus2.0).

### Molecular analysis

Following karyotypic identification, 10 swamp, 6 crossbred and 4 murrah buffaloes were selected for genomic study. The DNA was extracted from the blood samples using DNeasy Blood and Tissue Kit (Qiagen) according to manufacturer’s protocol. To amplify the mitochondrial D-loop region, four primers (Table 1) were designed based on the known *Bubalus bubalis* mtDNA sequences [14], positioned in the conserved tRNA-Pro and tRNA-Phe. The volume of reaction mixture used for optimization of DNA was 50 μl that consisted of 25 μl of polymerase chain reaction (PCR) Master Mix (Promega GoTaq Green Master Mix), 0.4 pmol of both forward and reverse primers and ~ 60 ng/ μl of extracted DNA. Amplification was done using Applied Bioscience Thermocycler following PCR profile; preliminary denaturation at 95 °C for 3 mins, followed by 35 cycles of 94 °C for 30 s, 58 °C for 1 min and 72 °C for 1 min. This was followed by a final extension period of 72 °C for 4 mins. PCR products were electrophoresed on 1% agarose gels and purified using the Macherey-Nagel NucleoSpin Gel and PCR Clean-up according to the manufacturer’s instructions. Purified DNA was directly sequenced using Applied Biosystems BigDye Terminator v3.1 cycle sequencing kit chemistry for both forward and reverse strands.

**Table 1:** Oligonucleotides used for DNA amplification and sequencing of the bubaline mitochondrial D-loop region

Name	sequences
H15773F	5′-ATA GCC CCA CTA CCA ACA CC-3’
L16371R	5′-TTA AGG GGA AAG AGT GGG CG-3’
H16231F	5′-ACC AGC AAC CCT TCA GAC AG-3’
L421R	5′-TTT TCA GTG CCT TGC TTT GGT-3’

The PCR products were used for sequencing (First Base, Malaysia). The sequences were edited and aligned using Geneious 11.1.12. All insertions/deletions in the alignment were used for multiple alignments. To demonstrate the phylogenetic clusters, the MEGA version 7 [15] was applied to construct maximum likelihood (ML) trees. Parameters used to construct the ML trees included using Hasegawa-Kishino-Yano model with 2000 bootstrapping replicates, Gamma distribution (G+) with 2 rate categories.

## Results

**Fig. 1:**
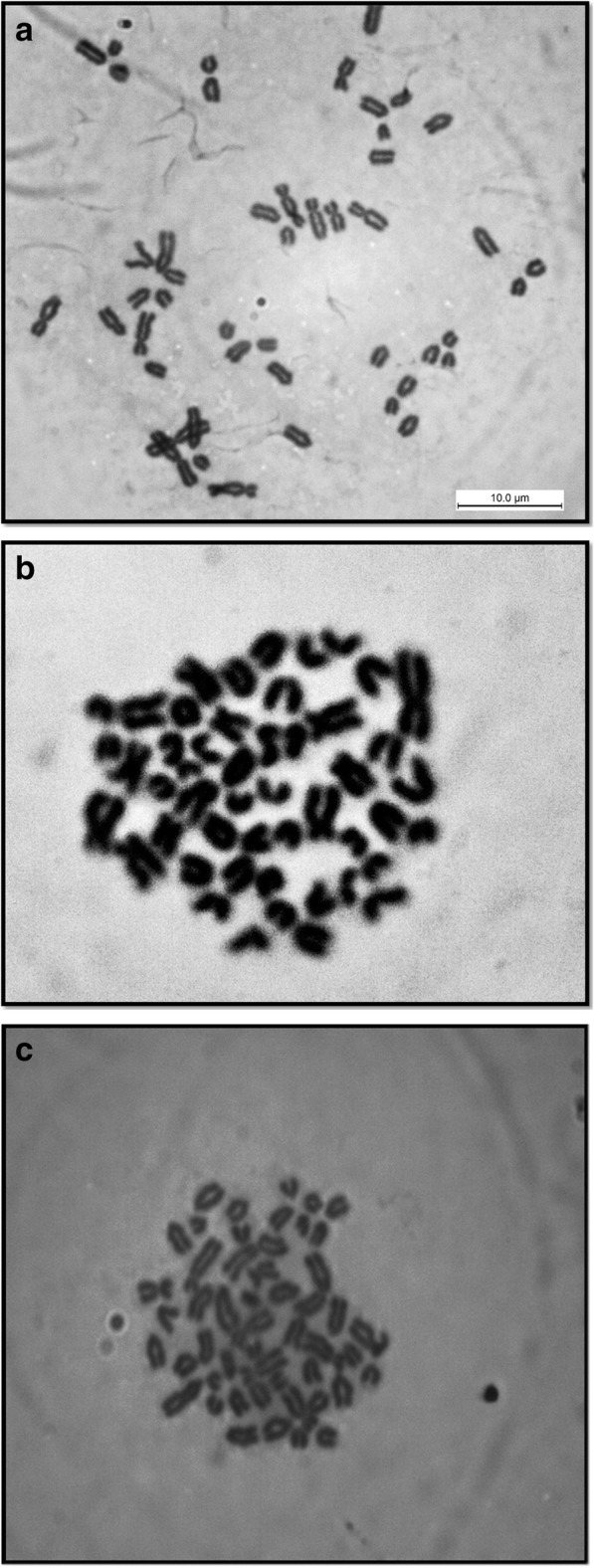
**a** Metaphase field exhibits 48 chromosomes showing female swamp buffalo (2n=48) by using giemsa staining method under 1000 magnification. **b** Metaphase field exhibits 49 chromosomes showing female crossbreed buffalo (2n=49) using giemsa staining method under 1000 magnification. **c** Metaphase field exhibits 50 chromosomes showing female murrah buffalo (2n=50) by using giemsa staining method under 1000 magnification

The number and shape of the chromosomes of each sub-species buffalo are shown in Fig. 1. From the 105 selected buffaloes, all 93 were identified as swamp buffaloes with 48 chromosomes (Fig. 1a), 7 were crossbreds with 49 chromosomes (Fig. 1b) and 5 were murrah buffaloes with 50 chromosomes (Fig. 1c). The 48 chromosomes of swamp buffaloes consisted of 1 metacentric and 4 submetacentric chromosomes and the remaining were acrocentric, including the sex chromosomes. The crossbreds were presented with 49 chromosomes with 1 metacentric, 4 submetacentric and 18 acrocentric chromosomes. However, murrah buffaloes had 5 metacentric chromosomes and the remaining 20 pairs were acrocentric, including the sex chromosomes.

**Fig. 2:**
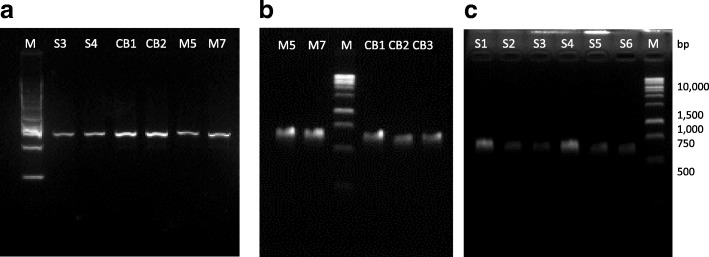
**a** Purified PCR product of primer H15773F and L16371R for swamp, crossbreed and murrah samples. The size is estimated around 500 bp. M represent 1 kb marker (Promega). **b** Purified PCR product of primer H16231F and L421R for murrah and crossbreed samples. The size is estimated around 500 bp. M represent 1 kb marker (Promega). **c** Purified PCR product of primer H16231F and L421R for swamp samples. The size is estimated around 500 bp. M represent 1 kb marker (Promega)

Polymerase chain reaction had successfully amplified the mitochondrial D-loop region of the buffalo samples. Both pairs of primers produced between ~ 500 to 750 bp of PCR products (Fig. 2a,b,c). The two sequences were aligned and combined to produce a 920 bp mitochondrial D-loop DNA. The mtDNA D-loop sequences had been deposited in the NCBI GenBank where the swamp (*n* = 10) accession numbers were MH746466-MH746475, the crossbreds (*n* = 6) accession numbers were MH746476-MH746481 and the murrah (*n* = 4) accession numbers were MH746482-MH746485. The D-loop regions of both swamp and crossbreds had slightly lower A + T content (58.28 ± 0.17% and 58.23 ± 0.15%) than the murrah buffaloes (59.48 ± 0.83%).

Comparing the DNA sequences of all 20 buffaloes (Table 2) revealed 10 different haplotypes with haplotype diversity of 0.8000 ± 0.089. The DNA sequence of the haplotype Swamp-3951 appeared with highest frequency (45%) and was shared by individuals from both swamp (*n* = 7) and crossbreds (*n* = 2). Within the murrah buffaloes, all four individuals revealed different haplotypes with haplotype diversity of 1.000 ± 0.177. The swamp buffaloes revealed three haplotypes with haplotype diversity of 0.5111 ± 0.164. Four haplotypes were observed among the crossbreds with haplotype diversity of 0.8667 ± 0.129.

**Table 2:** Comparison of the haplotype diversity (*h*), variable sites, parsimony informative sites and nucleotide diversity (Pi) among the swamp and crossbreed buffalo populations in Telupid and murrah buffalo population in Semenyih Farm, Selangor

Population	n	NHap	Haplotype diversity, *h*	Variable sites	Parsimony informative sites	Nucleotide diversity, Pi
Swamp Telupid	10	3	0.511 ± 0.164	6/936	4/939	0.00177
Crossbreed	6	4	0.867 ± 0.129	5/936	4/936	0.00269
Murrah Selangor	4	4	1.000 ± 0.177	64/936	18/936	0.03821
Overall	20	10	0.800 ± 0.089	72/936	67/936	0.01906

*n* = number of samples; *NHap* = number of haplotypes

Sequence characterization revealed 72 variable sites in which 67 were parsimony informative sites with sequence diversity of 0.01881. It appeared that swamp had the lowest sequence diversity of 0.00177, followed with the crossbreds (0.00269) and the highest in murrah with sequence diversity of 0.03811. Similarly, the swamp buffaloes showed lowest values for nucleotide and haplotype diversity while murrah buffaloes showed the highest values. This indicated that the murrah buffaloes used in this study were genetically more diversified than the swamp and crossbred buffaloes.

**Fig. 3:**
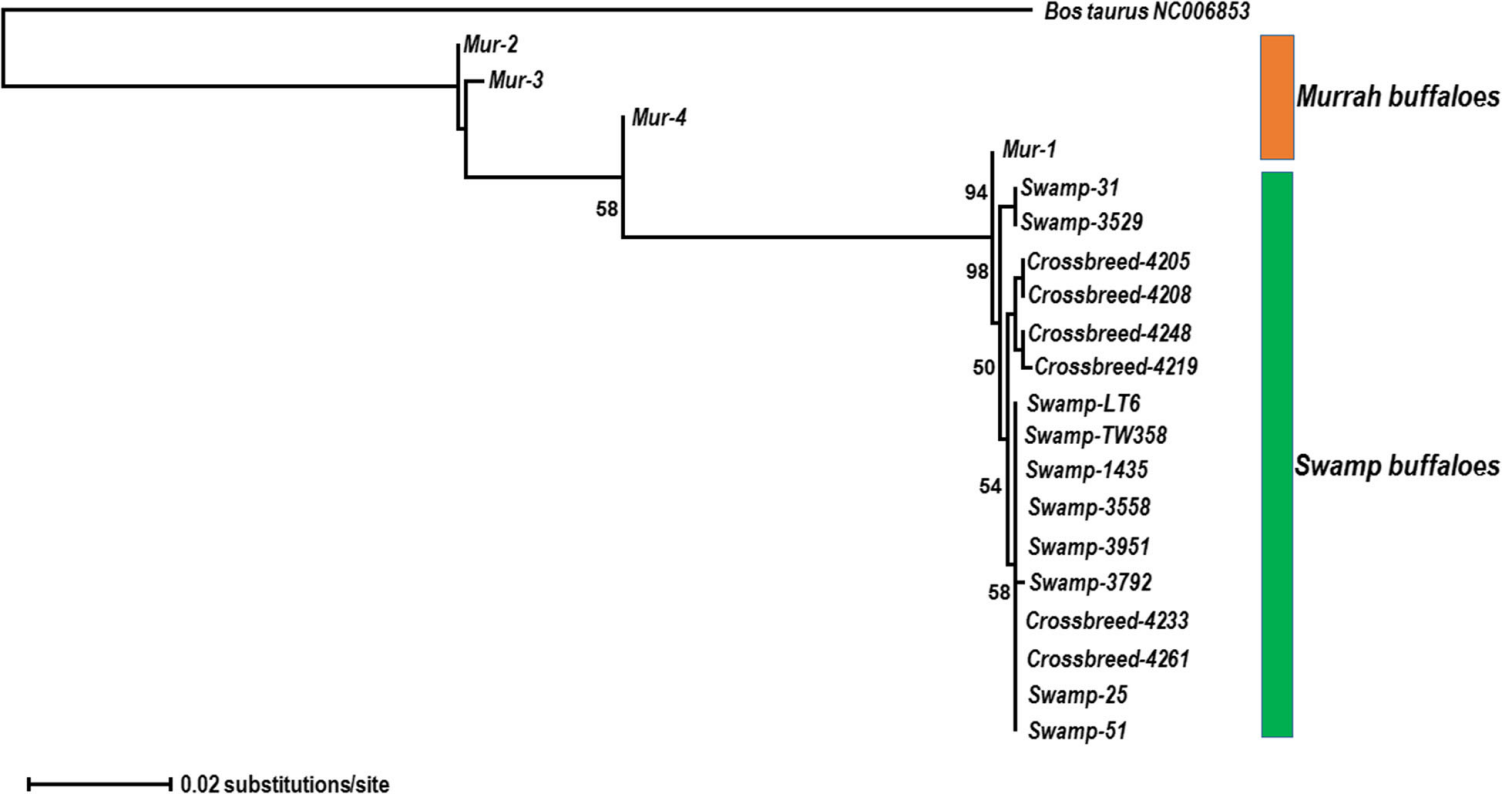
The maximum likelihood phylogram reconstructed by MEGA 7 from 10 swamp, 4 murrah and 6 crossbreed buffaloes of the mitochondrial D-loop region, rooted by *Bos taurus*

The phylogenetic tree revealed two distinct clades with extremely high posterior probability. Figure 3 shows the ability to distinguish between the swamp and the murrah buffaloes except for one murrah sample. The phylogenetic tree also revealed the fairly diversified murrah and much conserved swamp buffaloes. The 6 crossbreds were grouped in the same clade as swamp buffaloes, indicating the maternally descended from swamp buffalo.

## Discussion

The current study shows that the swamp and murrah buffaloes differ by two pairs of chromosomes. This is due to a tandem fusion translocation of chromosomes 4 and 9 that were observed in the murrah buffalo into chromosome 1 in the swamp buffalo [16, 17]. The crossbreds had 49 chromosomes. These were in line with findings from previous studies that reported 48 chromosomes for swamp buffaloes [9, 18] and 49 chromosomes for crossbreds [19]. Thus, karyotyping appeared to confirm the phenotypic identification of the swamp, murrah and crossbred buffaloes.

The mtDNA has been broadly used in phylogenetic study since it is inherited directly from the mother, thus no change was made in the copy that makes it a suitable tool to be used to study the relationship of individual in the same species or even in different species. Besides that, the high mutation rate in the mtDNA provides the best estimation of the evolutionary relationships between individuals [20–22]. This study grouped the crossbreds within the same clade as the swamp buffaloes, indicating the strong swamp maternal genetic since the farm is practicing crossbred between murrah males and swamp females [4, 5]. Based on the maximum likelihood tree constructed in this study, it was found that the murrah was the ancestor of swamp buffaloes when Mur-1 was observed to be more closely related to the swamp group. This is in line with a study by Kiersten et al.*,* (2004) [12] who reported that the two types of buffalo descended from a single domestication event some 5000 years ago. However, it contradicts the finding of Kumar et al.*,* (2007) [23] who concluded an independent domestication of murrah and swamp buffaloes based on the sequences of mtDNA D-loop region and the *cytochrome b* analyses.

The murrah buffalo in this study showed high variability when all 4 samples were placed in different subclades, even though they were sampled from the same farm. On the other hand, the swamp and crossbred buffaloes showed less diversity. The murrah buffaloes were most probably originated from various sourced following importations from Indian subcontinent throughout the twentieth Century [8]. The swamp buffalo on the other hand, is the indigenous buffalo in Malaysia and many countries of the Southeast Asia with little importations [24, 25].

The crossbreds were the product of crossbreeding between male murrah and female swamp buffaloes, producing 49 chromosomes. Therefore, the crossbreds were not reproductively isolated because they shared the conserved regions of the genes from the parents. In fact, the crossbreds performed better than the swamp buffaloes especially in the meat and milk productions [7].

## Conclusions

In conclusion, identification of the two buffalo sub-species and the crossbreds could be done phenotypically while confirmation was through karyotypic method, based on the number of chromosomes. In addition, analysis of mtDNA and the phylogenetic tree revealed that, the swamp buffaloes are genetically conserved and the crossbreds are dominantly swamp according to the maternal lineage using d-loop mtDNA. Thus, the combination of cytogenetic, phenotypic and mtDNA D-loop sequence analyses could be used to characterize the two buffalo subspecies and their crossbreds.

## Abbreviations

DVS: Department of Veterinary Services
G +: Gamma distribution
IACUC: Institutional for Animal Care and Use Committee
ML: Maximum likelihood
mtDNA: Mitochondrial DNA
Mur-1: Murrah 1
PCR: Polymerase Chain Reaction
